# Case Report: Endotracheal tube as life-saving tool to control an aortic rupture in intensive care unit

**DOI:** 10.12688/f1000research.128729.1

**Published:** 2022-12-21

**Authors:** Ciro Campanella, Mohannad Abbass, Umberto Marzi, Franco Masini, Salvatore Lentini

**Affiliations:** 1 Cardiac Surgical Department, Salam Centre for Cardiac Surgery, Khartoum, Sudan, Sudan

**Keywords:** Aortic rupture, deep sternal wound infection, cardiac surgery, haemorrhage

## Abstract

**Background:** Aortic rupture and suture dehiscence can complicate a cardiac operation, especially in case of infections of the surgical site. Such a complication can be life-threatening and require immediate surgical treatment.

**Case:** We report the case of a 13-year-old girl who suffered a sudden dehiscence of the aortic suture line in the context of deep sternal wound infection, while in the surgical intensive care unit after a double valve replacement. Control of bleeding was obtained by the insertion of an endotracheal tube into the ascending aorta and inflation of the tube cuff to plug the aortic bleeding point. The extracorporeal circulation was then established and under deep hypothermic arrest the defect was successfully repaired. The patient was discharged 14 days after surgery and reviewed at the outpatient clinic in good health.

**Conclusions:** An endotracheal tube can be used in cases of uncontrollable aortic bleeding as a life-saving tool to bridge the patient to adequate surgical treatment.

## Introduction

Sudden aortic rupture after cardiac surgery is a life-threatening complication that can lead to death. ^1^ Aortic rupture or dehiscence of an aortic suture can happen also as a consequence of post-cardiotomy deep sternal wound infections (DSWI), but reported outcomes of these cases are poor. ^2^ Diagnosis and treatment of such a complication can be challenging, especially if the sudden rupture happens outside the operating theatre where the establishment of an emergent surgical procedure is difficult. It is, thus, necessary to identify simple life-saving tools to control the aortic bleeding while bringing the patient to surgery. Herein, we report the case of a post-cardiotomy DSWI complicated by sudden dehiscence of the aortic suture controlled with the use of an endotracheal tube.

## Case report

This case report follows the CARE guidelines [8]. A 13-year-old girl from North Sudan was referred to our hospital for palpitations, fatigue, lower limb oedema and shortness of breath. Echocardiography showed severe aortic and mitral valve regurgitation with mildly reduced left ventricular function in the context of rheumatic heart disease. The patient underwent aortic and mitral valve replacement using a 19 mm SJM Regent mechanical prothesis (Abbott, Burlington, MA USA) in aortic position and a 27 mm SJM Regent mechanical prothesis (Abbott, Burlington, MA USA) in mitral position. The procedure was unremarkable, and the aorta was closed with a double layer technique.

The post-operative course was characterized by high fever (39.2°C) and inflammatory markers (white blood cells count: 15.75 10^3/uL; C-reactive protein: 248 mg/L) during the first post-operative day (POD). The patient was extubated on POD 4 but continued to be febrile with blood and wound cultures positive for *Klebsiella pneumoniae.* Chest x-rays showed signs of pneumonia. She was treated with empirical antibiotic therapy (amikacin 450 mg/150 ml once a day; ceftazidime 1.5 mg/50 ml three times a day) for 2 days and switched to piperacillin/tazobactam (4.5 g/50 ml continuous infusion at 4 ml/h) based on the antibiotic sensitivity. On POD 8 the sternal wound appeared swollen and oedematous with minimal discharge and 2 days later the patient was taken back to the operating theatre for wound re-exploration. The sternal wound was reopened and the diagnosis of DSWI was confirmed. Careful debridement was carried out. The sternum and skin were left open, swabs soaked in diluted chlorhexidine were left in place.

The wound rapidly improved in the subsequent 48 hours. On the day before the planned surgical closure, the patient developed a sudden bleeding through the drains. The swabs covering the heart were urgently removed in the intensive care unit (ICU) and active bleeding was noted from the aortotomy suture line. Digital pressure secured partial control, but the patient developed haemorrhagic shock.

## Surgical technique

**Figure 1.**
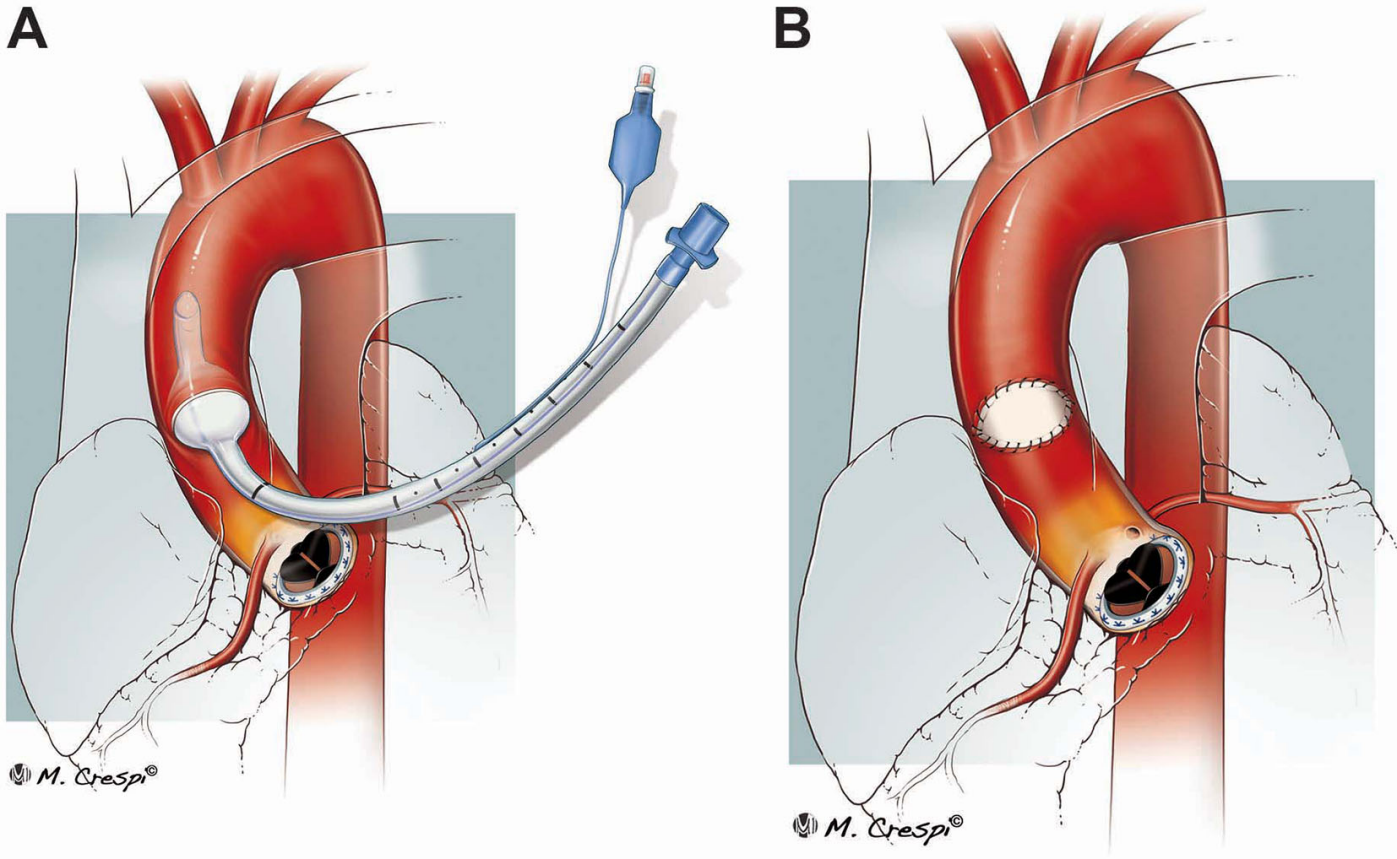
Endotracheal tube to control a tear in the ascending aorta. Representation of the main steps of the procedure to use an endotracheal tube as life-saving tool to control an aortic rupture in intensive care unit: temporary occlusion of the aortic tear with the use of an endotracheal tube (A) and subsequent closure of the tear with patch (B).

An attempt of placing a partial occluding clamp on the ascending aorta failed due to the rigid and inflamed aortic wall. Furthermore, a partial occluding clamp could have compromised the systemic and coronary blood flow. As an alternative strategy, a number 6 endotracheal tube (ENT Medical Instrument Co. LTD, China) was inserted into the aortic lumen through the aortic dehiscent suture line and its cuff was inflated with 10 ml of saline ( Figure 1A). By pulling the tube through the aortic opening, the inflated cuff accommodated itself inside the perimeter of the aortic opening securing a haemostatic seal and preventing occlusion of the aortic lumen and coronary ostia. It was then possible to see that the aortic suture line had cut through the aortic wall, generating a gap of around 2x2 cm.

The left femoral artery and the right atrium were cannulated, and cardiopulmonary bypass (CPB) was established. The patient was cooled to 24°C and deep hypothermic circulatory arrest (DHCA) with exsanguination (flow 500 ml/min) was established for 16 minutes (total CPB time 148 minutes). This allowed the avoidance of a crossclamp and cardioplegia administration, reducing the risks for further aortic wall damage. While on DHCA, the endotracheal cuff was deflated, and the tube removed. The patient was positioned in the Trendelenburg position and a pump sucker was inserted through the opening in the aortic wall and advanced toward the aortic arch. A pericardial patch of 2x1.5 cm was secured along the perimeter of the aortic dehiscence with a continuous 5/0 Prolene ( Figure 1B). The suture line was reinforced using surgical glue. Finally, the patient was weaned off CPB. Of note, the whole surgery was performed at the ICU bed.

The patient was left with the chest open for a further 48 hours. She remained stable through that period and underwent delayed chest closure. She was weaned from mechanical ventilation 72 hours after the last surgery and was discharged home 2 weeks later. She was reviewed at the outpatient clinic 4 weeks after the original procedure in good health.

## Discussion

Sudden aortic rupture is a catastrophic event that generally ends in patient death. ^1^^,^^2^ This case report illustrates not only the complications of a DSWI, but also the possibility to improvise major cardiac surgical procedure outside the operating theatre. To the best of our knowledge, this is the first report depicting the use of an endotracheal tube as haemostatic plug to control major bleeding from large vessels and one of the few reports of aortic rupture as consequence of DSWI.

DSWI is a severe complication after cardiac surgery, with a reported incidence ranging from 0.2 to 8.0% ^3^^,^^4^ and early mortality rates from 7.3% to 21.6%. ^5^^,^^6^ Patients with DSWI have significantly higher overall mortality, in-hospital mortality, follow-up mortality, and major adverse cardiovascular events compared with patients without DSWI. ^7^ While risk analyses and outcomes of DSWI have been widely investigated, the literature lacks detailed studies on causes of mortality. Moreover, very few reports are available regarding the consequences of a DSWI on aortic walls, especially after aortic surgery. In our case, the major bleeding occurred from the aortotomy site. The aggressive DSWI probably exacerbated an aortitis, which led to thinning and perforation of the weakest point of the aortic wall as previously described by Kim *et al.* ^2^

Sudden aortic rupture has a fatal outcome in most cases. ^1^^,^^2^ In our case a combination of “fortuitous” events allowed for a successful outcome. Firstly, the patient’s chest was already open and when the aortic suture line started bleeding it was sufficient to remove the swabs to confirm the diagnosis and apply a digital pressure. With a closed chest, time-lapse between the visualisation of the blood in the drainage bottles and the diagnosis would have resulted in the patient’s exsanguination. Furthermore, the decision to keep the patient at the ICU bed and call instead for the theatre staff and perfusionist personnel to help, proved extremely efficient allowing for emergency cannulation and CPB establishment in very few minutes. Finally, we feel that the unconventional use of an endotracheal tube proved vital to stop the massive bleeding and gave us the time to proceed to establish the CPB. Nevertheless, this is an anecdotical report on an unconventional technique to control aortic bleedings. As such, it is limited by a difficult reproducibility and by the off-label use of the endotracheal tube. Thus, this technique should be considered only as bail-out approach in case of lack of other conventional techniques.

In conclusion, this case illustrates that the management of a major uncommon complication in cardiac surgery is possible even in challenging logistical circumstances. However, further studies are required to clarify this.

## Consent

Written informed consent for publication of their clinical details was obtained from the father of the patient.

## Data Availability

All data underlying the results are available as part of the article and no additional source data are required. Figshare: CARE checklist for ‘Case Report: Endotracheal tube as life-saving tool to control an aortic rupture in intensive care unit’. https://doi.org/10.6084/m9.figshare.21617565. ^8^ Data are available under the terms of the Creative Commons Zero “No rights reserved” data waiver (CC0 1.0 Public domain dedication).
