# Supplementary material for: Case Report: Endotracheal tube as life-saving tool to control an aortic rupture in intensive care unit
Source: F1000Res. 2022 Dec 21;11:1545. [Version 1] doi: 10.12688/f1000research.128729.1 (PMC11549537; doi:10.12688/f1000research.128729.1)
Supplement: Peer review history

# Reviewer report: Reviewer response for version 1

**DOI:** 10.5256/f1000research.141348.r296771

**Published:** 2024-11-08

**Authors:** Jigisha Pujara

**Affiliations:** 1 U. N. Mehta Institute of Cardiology and Research Centre, Ahmedabad, Gujarat, India

This case report is an interesting case, in which authors have described the life saving technique for post Double valve replacement complication of sternal wound infection followed by aortic dehisence. Authors have described the innovative technique to control the bleeding by endotracheal tube cuff, to save the life and work as a bridge to definitive repair of aortic rupture. this case report can help other cardiac surgeons to do the similar management if they face same complication.

 Only suggestion to author is, kindly recheck the antbiotic dosages,which are not correct or typographical error> please recheck them and correct. (ideally dosage to be written as per kg dose).

Are enough details provided of any physical examination and diagnostic tests, treatment given and outcomes?

Yes

Is the case presented with sufficient detail to be useful for other practitioners?

Yes

Is sufficient discussion included of the importance of the findings and their relevance to future understanding of disease processes, diagnosis or treatment?

Yes

Is the background of the case’s history and progression described in sufficient detail?

Yes

Reviewer Expertise:

Cardiac Anesthesia related articles and critical care

I confirm that I have read this submission and believe that I have an appropriate level of expertise to confirm that it is of an acceptable scientific standard.

# Reviewer report: Reviewer response for version 1

**DOI:** 10.5256/f1000research.141348.r158587

**Published:** 2023-01-17

**Authors:** Justine Mafalda Ravaux

**Affiliations:** 1 CARIM, Maastricht, The Netherlands; 2 Universitair Ziekenhuis Leuven, Leuven, Belgium

Campanella and colleagues presented a single case report experience of an endotracheal tube used as a plug for managing an aortic rupture outside in an intensive care unit. The progression of the issue’s management is clearly described and the authors provided sufficient background in the field of interests. Moreover, details about diagnostic test, procedure (surgical technique, logistic…) are correctly mentioned in the manuscript. The discussion emphasizes the feasibility of the technique when performing high-risk interventions in a “hostile” environment, allowing other practitioners to consider such an option when dealing with a similar problem. The manuscript is overall well-written and no English revision is needed. If we think of the current intra-aortic balloon used for mini-invasive surgical procedures (Intraclude, Edwards Lifesciences Inc), this case report sounds a little bit like a “Back to Basics”, which may be helpful in a non-surgical environment. The authors should be congratulated for reporting such a kind of “home-made” solution, when the scientific community is globally focusing on the development of new complex devices and/or techniques, while the focus should be on the implementation of all these techniques worldwide, avoiding two-speed medicine. Herewith enclosed are my minor comments.

- Abstract, line 7. “ *(…)while in the surgical intensive care(…)*”. Please rephrase with “(…)occurring at the intensive care(…)”.
- Abstract, lines 15-17. I would emphasize that these techniques may especially be considered in a non-surgical environment.
- Introduction, line 7. I would add to the last sentence that this manoeuvre was performed at the intensive care unit.
- Case report, line 9. The authors mentioned positive wound culture *for Klebsiella Pneumonia*. Did the patient experience a leak from the sternal wound with pus or other sign of wound infection, like dehiscence of the wound? Please specify.
- Surgical technique. After placing the endotracheal tube to control the aortic bleeding at the intensive care unit, why did the team not transfer the patient into the operating room for further surgical management? Was the patient too hemodynamically unstable or was it easier (in the way, faster) to ask the whole team to come to the intensive care unit?

Are enough details provided of any physical examination and diagnostic tests, treatment given and outcomes?

Yes

Is the case presented with sufficient detail to be useful for other practitioners?

Yes

Is sufficient discussion included of the importance of the findings and their relevance to future understanding of disease processes, diagnosis or treatment?

Yes

Is the background of the case’s history and progression described in sufficient detail?

Yes

Reviewer Expertise:

Pacemaker implantation, rhythm disturbances, heart mechanical support, ECMO, coronary artery disease, aortic disease, sternal wound infection, mitral regurgitation

I confirm that I have read this submission and believe that I have an appropriate level of expertise to confirm that it is of an acceptable scientific standard.
